# Supplementary material for: AffyMAPSDetector: a software tool to characterize Affymetrix GeneChip™ expression arrays with respect to SNPs
Source: BMC Bioinformatics. 2007 Jul 30;8:276. doi: 10.1186/1471-2105-8-276 (PMC1959249; doi:10.1186/1471-2105-8-276)
Supplement: Additional file 13 — AffyMAPSDetector v1 source code. AffyMAPSDetector source code. [file 1471-2105-8-276-S13.zip › AffyMAPSDetector_v1_src/doc/affychip/snpdetector/model/event/StatusEvent.html]

StatusEvent


|  |  |  |  |
| --- | --- | --- | --- |
| **Overview** \| **Package** \| **Class** \| **Use** \| **Tree** \| **Deprecated** \| **Index** \| **Help** | | |  |
| PREV CLASS   NEXT CLASS | **FRAMES**    **NO FRAMES**     **All Classes** |  |  |
| SUMMARY: NESTED \| FIELD \| CONSTR \| METHOD | DETAIL: FIELD \| CONSTR \| METHOD |  |  |


---


## affychip.snpdetector.model.event Class StatusEvent

```
java.lang.Object
  java.util.EventObject
      affychip.snpdetector.model.event.StatusEvent
```

**All Implemented Interfaces:**: java.io.Serializable

---

public class **StatusEvent** extends java.util.EventObject

Title:

Description: It represents model specific event. An information processing
status event is generated on each SNP detection information update.

Copyright: Copyright (c) 2004

**Version:**
:   1.1

**Author:**
:   Dr. Sunita Kumari

**See Also:**: Serialized Form

---

**Field Summary**

**Fields inherited from class java.util.EventObject**

`source`


**Constructor Summary**

`StatusEvent(java.lang.Object src,
java.lang.String message)`

`StatusEvent(java.lang.Object src,
java.lang.String message,
java.awt.Color visualArtifact)`


|  |  |
| --- | --- |
| **Method Summary** | |
| `java.lang.String` | `getMessage()` |
| `java.awt.Color` | `getVisualArtifact()` |

**Methods inherited from class java.util.EventObject**

`getSource, toString`

**Methods inherited from class java.lang.Object**

`clone, equals, finalize, getClass, hashCode, notify, notifyAll, wait, wait, wait`

**Constructor Detail**

### StatusEvent

```
public StatusEvent(java.lang.Object src,
                   java.lang.String message)
```

---


### StatusEvent

```
public StatusEvent(java.lang.Object src,
                   java.lang.String message,
                   java.awt.Color visualArtifact)
```


**Method Detail**

### getMessage

```
public java.lang.String getMessage()
```

---


### getVisualArtifact

```
public java.awt.Color getVisualArtifact()
```


---


|  |  |  |  |
| --- | --- | --- | --- |
| **Overview** \| **Package** \| **Class** \| **Use** \| **Tree** \| **Deprecated** \| **Index** \| **Help** | | |  |
| PREV CLASS   NEXT CLASS | **FRAMES**    **NO FRAMES**     **All Classes** |  |  |
| SUMMARY: NESTED \| FIELD \| CONSTR \| METHOD | DETAIL: FIELD \| CONSTR \| METHOD |  |  |


---
